# Disturbance of neuron–microglia crosstalk mediated by GRP78 in Neuropsychiatric systemic lupus erythematosus mice

**DOI:** 10.1186/s12974-023-02832-8

**Published:** 2023-06-26

**Authors:** Jingyi Xu, Chunshu Yang, Siyuan Zeng, Xuejiao Wang, Pingting Yang, Ling Qin

**Affiliations:** 1 Department of Rheumatology and Immunology, The First Hospital of China Medical University, Shenyang, 110001 People’s Republic of China; 2 Department of 1st Cancer Institute, The First Hospital of China Medical University, Shenyang, 110001 People’s Republic of China; 3 Department of Physiology, School of Life Science, China Medical University, Shenyang, Liaoning Province 110122 People’s Republic of China

**Keywords:** Neuropsychiatric systemic lupus erythematosus, Glucose regulatory protein 78, Microglia, Spatial memory, Rapamycin

## Abstract

**Objectives:**

Neuropsychiatric systemic lupus erythematosus (NPSLE) is a serious phenotype of systemic lupus erythematosus (SLE). The disturbance of neuron–microglia crosstalk is recently revealed in many neuropsychiatric diseases but was not well studied in NPSLE. We found glucose regulatory protein 78 (GRP78), a marker of endoplasmic reticulum stress, was significantly increased in the cerebrospinal fluid (CSF) of our NPSLE cohort. We, therefore, investigated whether GRP78 can act as a mediator between the neuron–microglia crosstalk and is involved in the pathogenic process of NPSLE.

**Methods:**

Serum and CSF parameters were analyzed in 22 NPSLE patients and controls. Anti-DWEYS IgG was injected intravenously into mice to establish a model of NPSLE. Behavioral assessment, histopathological staining, RNA-seq analyses, and biochemical assays were performed to examine the neuro-immunological alterations in the mice. Rapamycin was intraperitoneally administered to define the therapeutic effect.

**Results:**

The level of GRP78 was elevated significantly in the CSF of the patients with NPSLE. An increase in GRP78 expression, accompanied by neuroinflammation and cognitive impairment, was also found in the brain tissues of the NPSLE model mice induced by anti-DWEYS IgG deposition on hippocampal neurons. In vitro experiments demonstrated that anti-DWEYS IgG could stimulate neurons to release GRP78, which activated microglia via TLR4/MyD88/NFκB pathway to produce more pro-inflammatory cytokines and promote migration and phagocytosis. Rapamycin ameliorated GRP78-inducing neuroinflammation and cognitive impairment in anti-DWEYS IgG-transferred mice.

**Conclusion:**

GRP78 acts as a pathogenic factor in neuropsychiatric disorders via interfering neuron–microglia crosstalk. Rapamycin may be a promising therapeutic candidate for NPSLE.

## Introduction

Systemic lupus erythematosus (SLE) is a chronic, autoimmune, and inflammatory disease with multi-system injuries, characterized by a breakdown of immune tolerance and the production of a broad spectrum of antibodies to self-antigens [1]. The central nervous system (CNS) disorder, neuropsychiatric SLE (NPSLE), is one of the most severe manifestations of the disease that has a heavy impact on patients’ functioning, quality of life, and disease outcome [2]. Although a variety of factors, including blood–brain barrier (BBB) impairment, autoantibody, vascular occlusion, and cytokine, have been suggested to associate with the development of NPSLE, its pathogenesis remains unclear. Recent research suggests that some autoantibodies, such as anti-ribosomal P protein antibodies and anti-dsDNA antibodies can directly bind to neurons through disrupted BBB, resulting in neuronal dysfunction and damage [3]. For example, a subset of anti-dsDNA antibodies cross-reacts with the N-methyl-D-aspartic acid receptor (NMDAR) by recognizing the 5-amino acid consensus sequence D/E W D/E Y S/G (DWEYS, for short) [4–8]. When BBB integrity is broken, these antibodies traverse the BBB, enter the hippocampus, increase the size of NMDAR-mediated excitatory postsynaptic potentials and promote excitotoxicity [8, 9].

On the other hand, microglia are also critical to the structural alterations of neurons in the anti-DWEYS antibody-mediated model [5, 8, 9]. There is growing evidence that the crosstalk between neurons and microglia, mediated by purinergic signaling, cytokines, neurotransmitters, and neuropeptides, plays a key role in surveillance, phagocytosis, modulation of synaptic transmission, and neural plasticity [10]. The disturbance of neuron–microglia crosstalk is recently revealed in many neuropsychiatric diseases including Alzheimer’s disease (AD), amyotrophic lateral sclerosis (ALS), and Parkinson’s disease (PD) [11]. However, the involvement of neuron–microglia crosstalk in NPSLE is still not well studied.

Endoplasmic reticulum (ER) stress is an imbalance between the protein folding capacity of the ER and the client protein load, which can be induced by hypoxia, nutrient deprivation, low pH, and metabolic disturbance [12]. Neurons are particularly sensitive to ER stress, which was found to be involved in the pathogenesis of many neurodegenerative diseases [12, 13]. For instance, Aβ oligomers can stimulate ER Ca^2+^ release through ryanodine receptors and IP3 receptors, which trigger neuronal ER stress, apoptosis, and mitochondrial fragmentation in AD [14–16]. It has been reported that exposure to SLE plasma or anti-dsDNA antibodies can induce ER stress in human endothelial or mesangial cells [17]. The involvement of ER stress in the neuropathology of NPSLE is worthy of further studies.

In this study, we first found an obvious elevation of glucose-regulated protein 78 (GRP78, a key marker of ER stress) level in the cerebrospinal fluid (CSF) of NPSLE patients. During ER stress, GRP78 dissociates from the ER membrane to aid in targeting misfolded and unfolded proteins for degradation, which limits ER stress and the potential toxicity of these proteins [12, 18, 19]. Meanwhile, GRP78 can also induce microglia to produce more cytokines and enhance phagocytosis [20]. We, therefore, hypothesize that pathogenic antibodies in SLE might bind to neurons and induce the production and release of GRP78, which activates microglia to produce pro-inflammatory cytokines and engulf synaptic proteins, in turn, aggravates neuronal damage. To testify to this, we transferred anti-DWEYS IgG into Balb/c mice, which bind to NMDAR of hippocampal neurons, and induce neuronal dysfunction and cognitive impairment similar to NPSLE patients [8, 9]. We examined the effect of anti-DWEYS IgG on the hippocampal neurons to produce GRP78 and the mechanism of GRP78-induced microglial activation in anti-DWEYS IgG-transferred mice. We further explored whether the application of rapamycin can reduce GRP78 production and ameliorate neuroinflammation and cognitive impairment. Our results highlight the important role of GRP78 in the neuron–microglia crosstalk and provide a potential therapeutic approach for NPSLE.

## Materials and methods

### Patients and samples

Neuropsychiatric manifestations were classified according to the ACR nomenclature and case definitions for NPSLE [21]. Patients hospitalized at First Affiliated Hospital of China Medical University from 2018 to 2021 were included. Primary headache patients excluded from CNS involvement by lumbar puncture were enrolled as the control group. All information about clinical symptoms and laboratory data was reviewed retrospectively using the patients' medical records. The protocol was approved by the Ethics Committee of First Affiliated Hospital of China Medical University (#AF-SOP-07-1.1-01), and informed consents were obtained from all the subjects. In all the cases, serum and CSF samples were obtained during the clinical assessment. All samples were stored at − 80 °C until further analyses were conducted.

### Antibodies and affinity chromatography

Anti-DWEYS IgG antibodies were obtained from rabbits immunized with the epitope containing the peptide DWEYSVWLSN [4, 6]. This peptide was also used for affinity chromatography purification of anti-DWEYS antibodies, as previously described [22]. The control (Ctl) IgG was affinity purified from normal rabbits.

### Animals and treatments

BALB/c male mice were obtained from HFK Bioscience (Beijing, China) aged 6–8 weeks. All animals were housed two to four per cage under conventional laboratory conditions (12 h/12 h light/dark cycle, 22 °C) with ad libitum access to food and water throughout the experiments. Experimenters handled the mice on alternate days during the week preceding the first behavioral test. Experimenters were blind to the mouse treatments during testing and behavioral scoring. All laboratory procedures were carried out following the policies and procedures detailed in the Guidelines for The Care and Use of Laboratory Animals of the National Institutes of Health and approved by the Animal Protection and Use Committee of China Medical University (No. Kt2020113).

After a 1-week habitual period, the mice received an intravenous injection of 2 mg anti-DWEYS IgG in 100 μl of PBS or Isotype IgG (Ctl) six times in 48 h intervals. The animals for each group were chosen randomly, based on litter. Intraperitoneal injection of 3 mg/kg LPS was conducted at 15 min after the first twice IgG injection to destroy the integrity of BBB [23, 24]. Behavioral assessments were conducted two days after the last injection of IgG (12 days after the BBB breach by LPS). Then, the animals were perfused by cold PBS under anesthesia. Brains were extracted, one half were stored in a hypothermic environment for RNA sequencing, western blot (WB), and enzyme-linked immunosorbent assay (ELISA), and the other half were fixed in the paraformaldehyde (PFA), preparing for the frozen section.

### Immunohistochemistry

The brain tissue was cryosectioned coronally in 15-μm-thick slices. The sections were washed with PBS, and blocked with 5% bovine serum albumin (BSA) with 0.3% Triton X-100 for 1 h at room temperature and then incubated overnight at 4 °C with the following primary antibodies diluted in PBS with 2% BSA and 0.3% Triton X-100: anti-NeuN (1:500, Abcam Inc., USA), anti-Iba-1 (1:400, Abcam), anti-GFAP (1:500, Abcam), anti-GRP78 (1:200, ProteinTech USA and ImmunoWay USA), or anti-PSD-95 (1:500, Abcam).

After three washes, secondary goat anti-rabbit Alexa Fluor 488 IgG (1:400, Thermo Fisher Scientific, USA) and/or goat anti-mouse Alexa Fluor 546 IgG (1:400, Thermo Fisher Scientific) were added at 1:400 for 2 h at room temperature. Sections underwent additional washes and were mounted with Antifade Mounting Medium (Beyotime, China) and cover-slipped. Nexcope NIB410 microscope system (NEXCOPE, USA) was used for Image acquisition and ImageJ software (NIH/ImageJ, Bethesda, USA) was used for analysis.

Immunofluorescence staining of anti-TLR4 (1:400, ImmunoWay) antibody anti-iNOS antibody (1:500, Proteintech), and anti-CD206 antibody (1:500, Proteintech) for cultured primary microglia and BV2 cells was performed similarly.

### Cytokine assay

Hippocampi were extracted from perfused brains and a lysate was made by sonication using a 20:1 ratio of lysis buffer to brain tissue followed by centrifugation (10,000 rpm, 30 min, 4 °C). Cytokines [interleukin (IL)-6, tumor necrosis factor (TNF)-α, and IL-1β] in lysate and cell culture supernatant were analyzed by ELISA kits (MULTI SCIENCES, China) according to the manufacturer’s instructions.

### Object place memory (OPM) task

Mice were transported inside their home cages into the darkened experimental room and placed in the empty chamber, for 4 sessions (2 per day) of 15 min. On the 3rd day, mice underwent OPM task in the following sequence: empty chamber (10 min), home cage (10 min), sample phase in which the chamber had two objects located at the center of the northwest and northeast sectors (10 min), home cage (10 min), choice phase in which the chamber had the same objects but one of them was moved from northeast to the center of the southeast sector (10 min). The number of visits and the times spent exploring each object on sample phase and choice phase were used for statistical comparisons. For sample phase, an exploration ratio was defined as the time exploring the right object minus the time exploring the left object over the sum of the times exploring both objects. For choice phase, an OPM ratio was defined as the time exploring the moved object minus the time exploring the stable object over the sum of the times exploring both objects.

### Rapamycin treatment

Rapamycin (MedChemExpress, China) was dissolved in dimethyl sulfoxide (DMSO) at 25 mg/ml before administration. Animals received rapamycin at a dose of 1.5 mg/kg per day through intraperitoneal injection or received DMSO only. The treatment started from two days before to the last day of anti-DWEYS IgG injection.

### RNA sequencing analysis

Total RNA of was isolated using the Trizol Reagent, and then the concentration, quality, and integrity of which were detected by a NanoDrop spectrophotometer (Thermo Scientific, USA). Sequencing libraries were prepared using the TruSeq RNA Sample Preparation Kit (Illumina, USA). The library fragments were purified using the AMPure XP system (Beckman Coulter, USA) to screen out cDNA fragments of a preferred length of 200 bp. The Illumina PCR Primer Cocktail was used to selectively enrich the DNA fragments with ligated adaptor molecules on both ends after 15 cycles of PCR reaction. After purification (AMPure XP system), the products were quantified using the Agilent high-sensitivity DNA assay on a Bioanalyzer 2100 system (Agilent, USA). The sequencing library was then sequenced on a Novaseq PE150 System (Illumina) by Shanghai Personal Biotechnology Cp. Ltd. Using TopHat2 upgrade HISAT2 (http://ccb.jhu.edu/software/hisat2/index.shtml) software of the filtered Reads alignment to reference genome, sequence Mapping rate higher than 90%. Reads were uniformly distributed in all expressed genes. HTSeq was used to statistically compare the Read Count value of each gene as the original expression amount of the gene. We adopt edgeR for differences in gene expression analysis, and significance *p*-value < 0.05 was used as screening conditions for differentially expressed genes (DEGs). The volcano map of differentially expressed genes was plotted by the ggplots2 software package in R language. The DEGs were analyzed Kyoto Encyclopedia of Genes and Genomes (KEGG) pathway analysis (www.genome.jp/kegg/).

### Cell culture and viability assay

Mouse primary microglia and the immortal mouse microglial cell line (BV2), which are an established cell line used to study microglia responses [25], were used for in vitro experiments. Primary microglia were isolated from cultures of mixed cortical cells as described previously [26]. In brief, cortices from P1 mice were isolated and digested with a solution containing 0.125% trypsin and 1.5 mg/ml DNAse for 10 min at 37 °C. Then the mixture was filtered by a cell filter with a diameter of 70 μm. Cells from four pups were plated onto poly-d-lysine (PDL)-coated 75-cm^2^ tissue culture flasks. The culture medium (DMEM, 10% FBS, and 1% Pen/Strep) was changed on day 2. After 7–9 days in culture, the primary microglia were removed by the addition of 12 mM lidocaine (Sigma-Aldrich) and orbital shaking (180 rpm) for 20 min at 37 °C. The cells were centrifuged (350 g), and the pellet was resuspended in complete medium and plated onto PDL-coated tissue culture dishes. Microglia cell cultures were > 95% pure as determined with Iba-1. BV2 and neuro-2a(N2a) cells were routinely passaged in DMEM, 10% FBS, and 1% Pen/Strep.

Cell viability was determined by the Cell Counting Kit-8 (CCK-8, MCE) method. The cells were inoculated and treated with different concentrations of GRP78 for 24 h. CCK-8 solution (10 μl per 100 μl of medium in each well) was added, the plates were incubated at 37 °C for 30–60 min, and the absorbance at 450 nm was read on a SpectraMax Plus 384 microplate reader (Molecular Devices Corporation, USA).

### Quantitative real-time polymerase chain reaction (qRT-PCR)

Total RNA was extracted using the Purelink RNA microkit (Thermo Fisher Scientific) and cDNA was synthesized using a cDNA synthesis kit (Takara, Japan). The qRT-PCR was performed on a Roche 480 real-time PCR system using GAPDH as an internal control. The PCR program consisted of an initial denaturation step at 94 °C for 2 min, followed by 38 cycles of denaturation at 94 °C for 15 s, annealing at 58 °C for 30 s, and extension at 72 °C for 1 min. Primer (Sangon Biotech, China) sequences are listed in Table 1.

**Table 1:** Primer sequences used in qRT-PCR

Primer	Forward	Reverse
GRP78	ACTTGGGGACCACCTATTCCT	ATCGCCAATCAGACGCTCC
GAPDH	GTCGGTGTGAACGGATTTGG	GACTCCACGACATACTCAGC

### Western blotting

Hippocampi were homogenized on ice in 2% SDS, 95 mM NaCl, 25 mM Tris, pH 7.4, 10 mM EDTA, and protease inhibitor mixture (Roche, Switzerland). After centrifugation, extracts were used for Western blot. Extracts from brains, as well as cultured BV2 and N2a cells, were run on reducing SDS/PAGE gels, transferred to PVDF membrane (EMD Millipore), incubated overnight at 4 °C in primary antibody [rabbit anti-TLR4 (Wanleibio, China); rabbit anti-MyD88 (Abcam); rabbit anti-phospho-NF-κB p65 (Ser536) (Cell Signaling Technology, USA); rabbit anti-NF-κB p65 Rabbit mAb (Cell Signaling Technology); rabbit anti-GRP78 (ProteinTech)], and then incubated with an appropriate HRP-conjugated secondary antibody. Blots were developed with high-intensity chemiluminescence Western blotting substrate (Tanon, China) and captured by Tannon 5200Multi Imager (Tannon). Protein levels were quantified using densitometry with ImageJ software. Western blot results for brain protein extracts were normalized to actin.

### Flow cytometry

Cell surface staining was performed with FITC-labeled anti-MHC II (BioLegend, USA) and PE-labeled CD206 antibodies (BioLegend) for 20–30 min. The background was assessed using Isotype control antibodies. Cells were analyzed using BD FACScan flow cytometry (Becton, Dickinson and Company, USA) and FlowJo software (version 7.6.1, Tree Star, Ashland, USA).

### Transwell migration assay

BV2 cells (5 × 10^4^ cells/well) suspended in serum-free DMEM were seeded into the transwell inserts (3422, Corning costar, USA). After incubation at 37 °C for 24 h, the lower chamber was filled with serum-free medium with or without a range of concentrations of GRP78 (1 μg/ml or 5 μg/ml) or TAK-242 (400 nM). After 24 h of treatment, the un-migrated cells were gently removed, and the migrated cells were fixed with 4% PFA for 20 min, permeated with methanol for 10 min, and then stained with 0.5% crystal violet for 10 min. The migrated cells were counted by ImageJ software. Migrated cells were quantified by counting the number of migrated cells in at least three random fields of view.

### Phagocytosis assay

Phagocytosis assay was performed using a pHrodo™ Red Zymosan Bioparticles kit (Invitrogen, USA). The Zymosan particles are nonfluorescent at neutral pH but emit strong fluorescence in an acidic environment after phagocytosis. Primary microglia were co-cultured 2 h at 37 °C with Zymosan particles, according to the manufacturer’s instructions.

### Statistical analysis

We used SPSS 23 software (IBM Inc., USA) and GraphPad software (GraphPad Prism v8.0, GraphPad Software Inc., USA) for statistical comparisons. Data were presented as mean ± SEM from three or more independent experiments. ANOVA, Student’s *t* test, and non-parametric tests, such as Kruskal–Wallis ANOVA and Kolmogorov–Smirnov test, were used as indicated in the text. *p* < 0.05 was considered statistically significant.

## Results

### Composition of the study groups

Matched serum and CSF were collected from 22 NPSLE patients and 22 controls (primary headache patients without definite CNS involvement). The demographic and clinical parameters are summarized in Table 2.

**Table 2:** Demographic and clinical characteristics of NPSLE patients and controls

Variable	Category	NPSLE (*n* = 22)	Control (*n* = 22)
Demographic variables	Age, years	26 (18–40)	27 (19.75–48)
	Gender (male/female)	3/19	13/9
	SLE duration, month	3.5 (0.5–12.0)	–
	SLEDAI-2 K, median (range)	16 (13–39)	–
Serum laboratory	IgG	17.30 (8.93–33.41)*	12.28 (10.68–13.77)
	IgM	1.26(0.65–2.22)	1.64 (0.92–2.75)
	IgA	2.31 (1.04–2.92)	2.59(1.38–3.41)
	C3, median (range),g/dL	0.53 (0.18–0.77)*	0.94 (0.71–1.30)
	C4, median (range),g/dL	0.06 (0.03–0.16)*	0.22 (0.11–0.37)
	CRP, median (range), mg/L	5.46(3.50–34.55)*	2.32 (1.13–4.03)
	ANA	22/22(100%)	0/22
	anti-dsDNA	12/22(54.6%)	0/22
	anti-ribosomal P	14/22(63.6%)	0/22
	ACL/β-GP1/LA	7/22 (31.9%)	0/22
Neuropsychiatric manifestations	Seizure disorders, *n* (%)	5 (22.7%)	–
	Acute confusional state, *n* (%)	7 (31.8%)	–
	Cognitive impairment, *n* (%)	5 (22.7%)	–
	Cerebrovascular disease^#^, *n* (%)	1 (4.5%)	
	Myelopathy, *n* (%)	2 (9.1%)	
	Meningitis, *n* (%)	1 (4.5%)	
	Psychosis, *n* (%)	1 (4.5%)	–
Routine parameters in CSF	Pressure (mmHg)	185 (90–220)	110 (85–160)
	Protein (mg/L)	843 (617–1339)	229 (196–307)
	Glucose (mmol/L)	3.25 (2–4.5)	3.20 (2–4.0)
	Chlorine (mmol/L)	122 (114–129)	123 (120–131)
	Cell counts (10 ^6^ / L)	8.5 (2–47)	4 (1–9)

Data are presented as median (interquartile range). *Significantly different between NPSLE patients and Controls (*p* < 0.01, Mann–Whitney U-test). ^#^ not including microangiopathy

### GRP78 level in CSF and sera

**Fig. 1:**
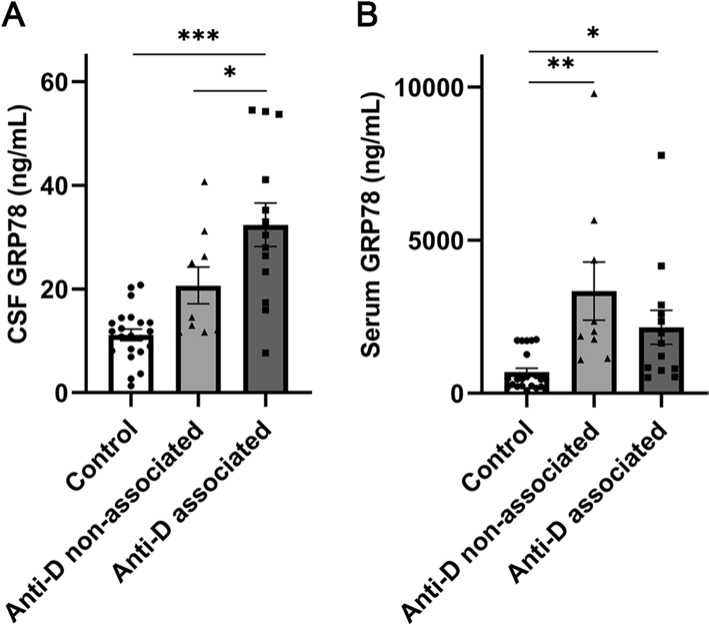
Comparison of GRP78 levels in CSF and serum among Anti-D associated and non-associated NPSLE and Control groups. **A** Levels of GRP78 in CSF among the three groups. **B** Levels of GRP78 in serum among the three groups. Data are mean ± SEM. *p < 0.05, **p < 0.01, ***p < 0.001; one-way ANOVA and Tukey’s multiple comparisons test

As acute confusional state, cognitive impairment, and psychosis have been associated with the anti-DWEYS antibody [27], we divided the subjects into three groups: control, and NPSLE patients with or without neuropsychiatric manifestations associated with anti-DWEYS antibody (Anti-D associated or non-associated NPSLE). Neuropsychiatric manifestations associated with anti-DWEYS antibody included acute confusional state, cognitive impairment, and psychosis. We found that the level of GRP78 in the CSF of patients with Anti-D associated NPSLE was significantly higher than in the other two groups [30.39 (20.34–47.41) vs. 11.39 (8.00–13.76) ng/ml, Tukey’s multiple comparisons test, *p* < 0.001 and 30.39 (20.34- 47.41) vs. 14.57 (11.88–28.75) ng/ml, Tukey’s multiple comparisons test, *p* < 0.05] (Fig. 1A). In contrast, there was no significant difference in CSF GRP78 levels between Anti-D non-associated NPSLE and the control (Tukey’s multiple comparisons test, *p* > 0.05) (Fig. 1A). Additionally, we observed a significant increase in serum GRP78 levels in both Anti-D associated NPSLE and Anti-D non-associated NPSLE compared to the control [1634.50 (774.29–2736.57) vs. 461.63 (224.47–1381.35) ng/ml, Tukey’s multiple comparisons test, *p* < 0.05 and 2023.87 (1454.51–5001.33) vs. 461.63 (224.47–1381.35) ng/ml, Tukey’s multiple comparisons test, *p* < 0.01], with no significant difference between the two NPSLE groups (*p* > 0.05) (Fig. 1B). Furthermore, there is no significant correlation between the GRP78 levels of CSF and sera in NPSLE patients (Spearman rho = − 0.190, *p* = 0.397).

### Deposition of anti-DWEYS IgG in the hippocampus causes microglial activation, inflammatory cytokine release, and spatial memory impairment in mice

**Fig. 2:**
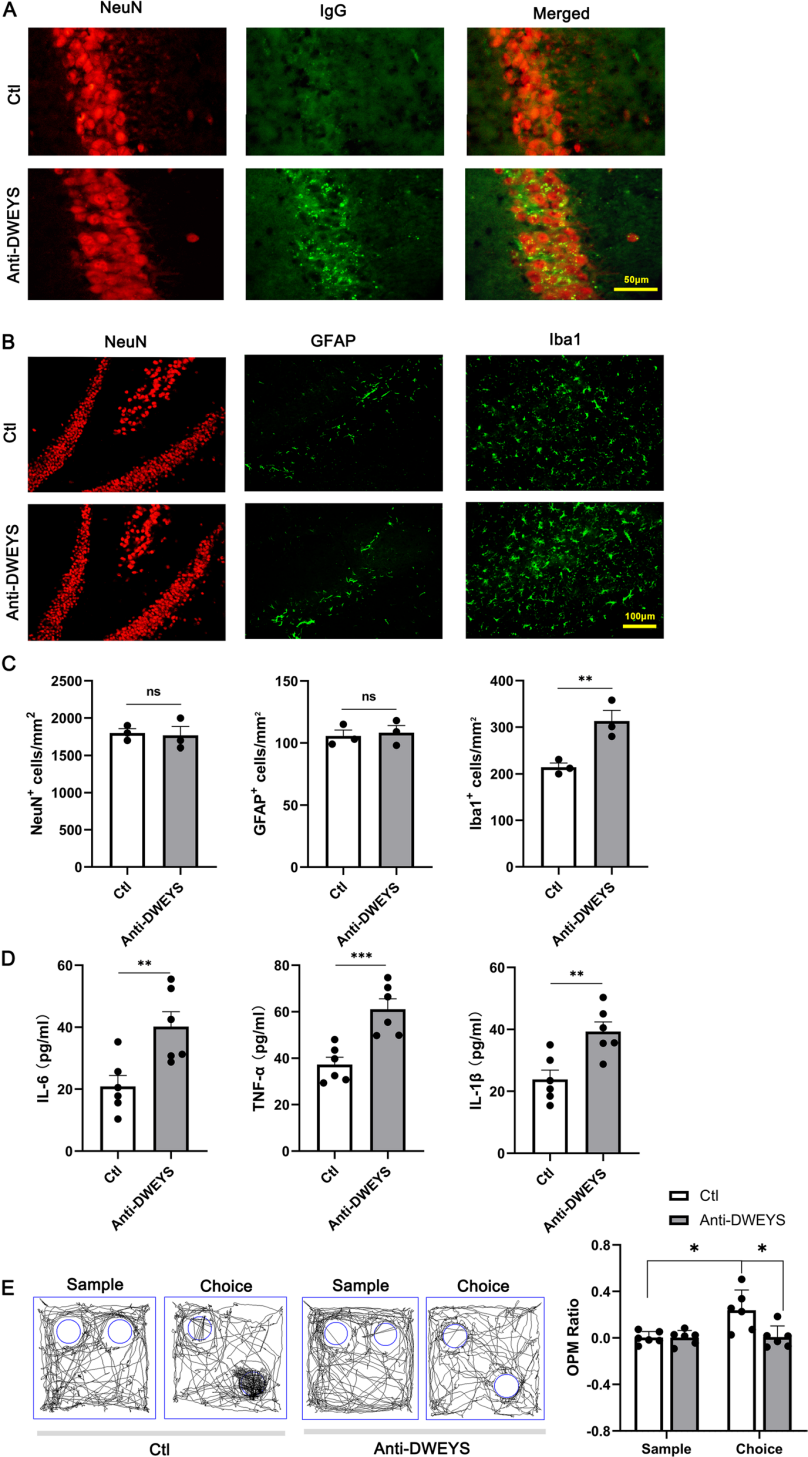
Anti-DWEYS induced-neuroinflammation in the hippocampus and spatial memory impairment. **A** Representative hippocampal sections from Ctl and anti-DWEYS mice that were stained for NeuN (red) and IgG (green). **B** Representative immunofluorescent staining for NeuN, Iba-1, and GFAP. **C** Quantification of NeuN^+^, Iba-1^+^, and GFAP^+^ cells of Ctl and Anti-DWEYS mice (*n* = 3). Data are mean ± SEM. **D** Comparisons of inflammatory cytokines IL-6, TNF-α, and IL-1β in the brain homogenates of Ctl and Anti-DWEYS mice (*n* = 3). **E** Left, representative track plots in OPM task in a Ctl and anti-DWEYS mice. Circles represent the locations of the objects. Right, OPM ratio showing that Ctl mice explored the moved object preferentially, while anti-DWEYS mice did not (*n* = 6). Data are mean ± SEM. ns, non-significant, *p < 0.05, **p < 0.01; ***p < 0.001; *t* test

We further conducted animal experiments to examine whether anti-DWEYS IgG can lead to the elevation of GRP78 in CSF and how the neuro-immunological homeostasis is disrupted. For this, we intravenously injected anti-DWEYS IgG into BBB-disrupted BALB/c mice (anti-DWEYS mice) to mimic clinical NPSLE. Isotype IgG was injected into the control mice (Ctl). We performed immunohistological staining of neuronal nuclear antigen (NeuN, a neural marker), ionized calcium-binding adapter molecule 1 (Iba-1, a microglia marker), and glial fibrillary acidic protein (GFAP, an astrocyte marker) on the brain slices of the hippocampus. Consistent with previous results [8, 9], anti-DWEYS IgG was observed to bind to NeuN^+^ cells in the hippocampus (Fig. 2A).

There was no significant difference in the count of neuron (NeuN^+^) and astrocyte (GFAP^+^) between anti-DWEYS and Ctl mice (Fig. 2B and C). However, the count of microglia (Iba-1^+^) was increased in the anti-DWEYS mice, in which the microglia were activated, showing retracted, thick processes and large irregular cell bodies. And enzyme-linked immunosorbent assays (ELISAs) revealed that the level of IL-6, TNF-α, and IL-1β was elevated in the hippocampus tissues of anti-DWEYS mice (Fig. 2D). The behavioral results of OPM task showed that the Ctl mice preferred to explore the moved object, whereas the anti-DWEYS mice did not, indicating that transfer of anti-DWEYS IgG disrupted spatial memory of mice (Fig. 2E).

### Anti-DWEYS IgG promotes protein processing in endoplasmic reticulum including GRP78 expression in hippocampal neurons

**Fig. 3:**
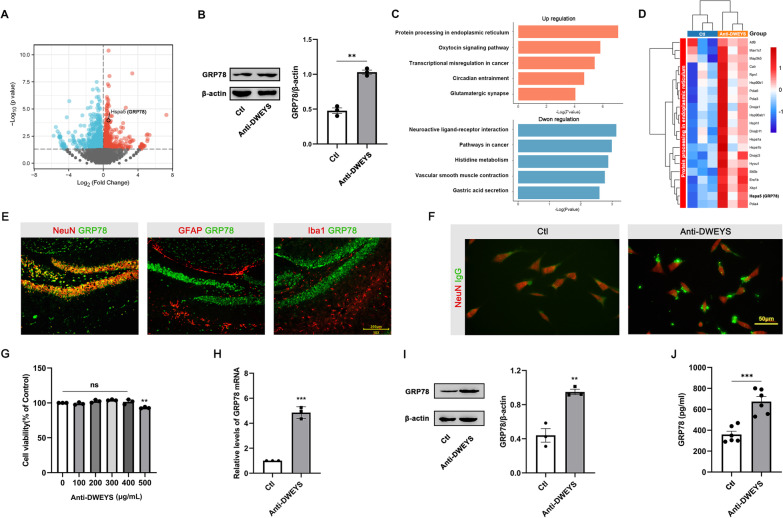
Divergent transcriptomic profiles and expression of GRP78 in the hippocampus of anti-DWEYS and Ctl mice. **A** Volcano plot showing the DEGs between anti-DWEYS and Ctl group. The horizontal dotted line corresponds to a p of 0.05. Down-regulated genes are shown in blue dots and up-regulated in red. **B** Left, western blot showing GRP78 expression levels in hippocampus of anti-DWEYS and Ctl mice. Right, densitometric quantification of GRP78 in different groups (*n* = 3). **C** KEGG pathway analysis of DEGs in up-regulated and down-regulated expression. **D** Heatmap showing the expression patterns of up-regulated DEGs in “protein processing in endoplasmic reticulum” pathway. **E** Double staining shows that GRP78 (green) is co-localized with NeuN (red, left), but not with GFAP (red, middle), or Iba1 (red, right). **F** Representative immunofluorescence staining for NeuN (red) and IgG (green) in N2a cells under anti-DWEYS or Isotype IgG stimulation. **G** Cell viability determined by CCK-8 assay (*n* = 3). Data are mean ± SEM. ns, non-significant; **p < 0.01; One-way ANOVA and Dunnett’s multiple comparisons test. **H** Quantitative RT-PCR analysis of GRP78 in N2a cell lysate (*n* = 3). ***p < 0.001, *t*-test. **I** Comparisons of GRP78 protein levels in the lysate of N2a cells after being stimulated by anti-DWEYS or Isotype IgG. **p < 0.01, *t*-test. **J** Comparisons of GRP78 levels in the supernatant of N2a cells after stimulated by anti-DWEYS or Isotype IgG. ***p < 0.001, *t*-test

We further performed transcriptome RNA sequencing (RNA-Seq) analysis on the hippocampus samples to assess the effects of anti-DWEYS IgG on gene expression. We selected differentially expressed genes (DEGs) with *p* < 0.05 and |fold change|> 1. One thousand five hundred and thirty-eight genes were deemed as DEGs for comparison between anti-DWEYS and Ctl groups (Fig. 3A). Of them, 756 genes were up-regulated including GRP78 (Hspa5), and 781 were down-regulated. Western blot analysis confirmed an increase of GRP78 protein level in the hippocampus tissue of anti-DWEYS mice (Fig. 3B). KEGG pathway analysis revealed that the most significant pathway enriched by up-regulated DEGs was the “protein processing in endoplasmic reticulum” (04141) pathway (Fig. 3C). Figure 3D shows the expression levels of 26 DEGs in “protein processing in endoplasmic reticulum” pathway including GRP78, suggesting that anti-DWEYS IgG may upregulate GRP78 expression involved in ER stress.

To determine the cell type responsible for the signal of GRP78, we performed double immunofluorescence staining of GRP78 with NeuN, GFAP, or Iba-1 in the hippocampus of anti-DWEYS mice. GRP78 co-localized well with NeuN and, to a lesser extent, with GFAP and Iba1, indicating that GRP78 was predominantly expressed by neurons (Fig. 3E). Next, we performed in vitro experiments to testify whether anti-DWEYS IgG can induce neurons to release GRP78. Mouse neuroblastoma neuro-2a (N2a) cells, which have commonly been used for the studies of hippocampal neurons [28], were treated with anti-DWEYS or isotype IgG (Ctl) and performed immunofluorescence assays to confirm the binding of the antibody to the cells (Fig. 3F). The cell viability of N2a cells was measured after treatment for 24 h using cell counting Kit-8 assays. Low-dose anti-DWEYS IgG (< 400 μg/ml) had no effect on cell viability (Fig. 3G). We also measured the expression levels of GRP78 mRNA and protein in cell lysates obtained from N2a cells treated with anti-DWEYS or isotype IgG. Our results showed that the expression levels of both GRP78 mRNA (Fig. 3H, t-test, *p* < 0.001) and protein (Fig. 3I, t-test, *p* < 0.01) were significantly elevated in the anti-DWEYS group compared to Ctl. Furthermore, we found that the level of GRP78 in the culture medium of the anti-DWEYS IgG-treated group was significantly higher than that of the isotype IgG-treated group (Fig. 3J, t-test, *p* < 0.001). Taken together, these results suggested that anti-DWEYS IgG could induce hippocampus neurons to express and release GRP78.

### GRP78 activates microglia via TLR4/MyD88/NFκB p65 pathway

**Fig. 4:**
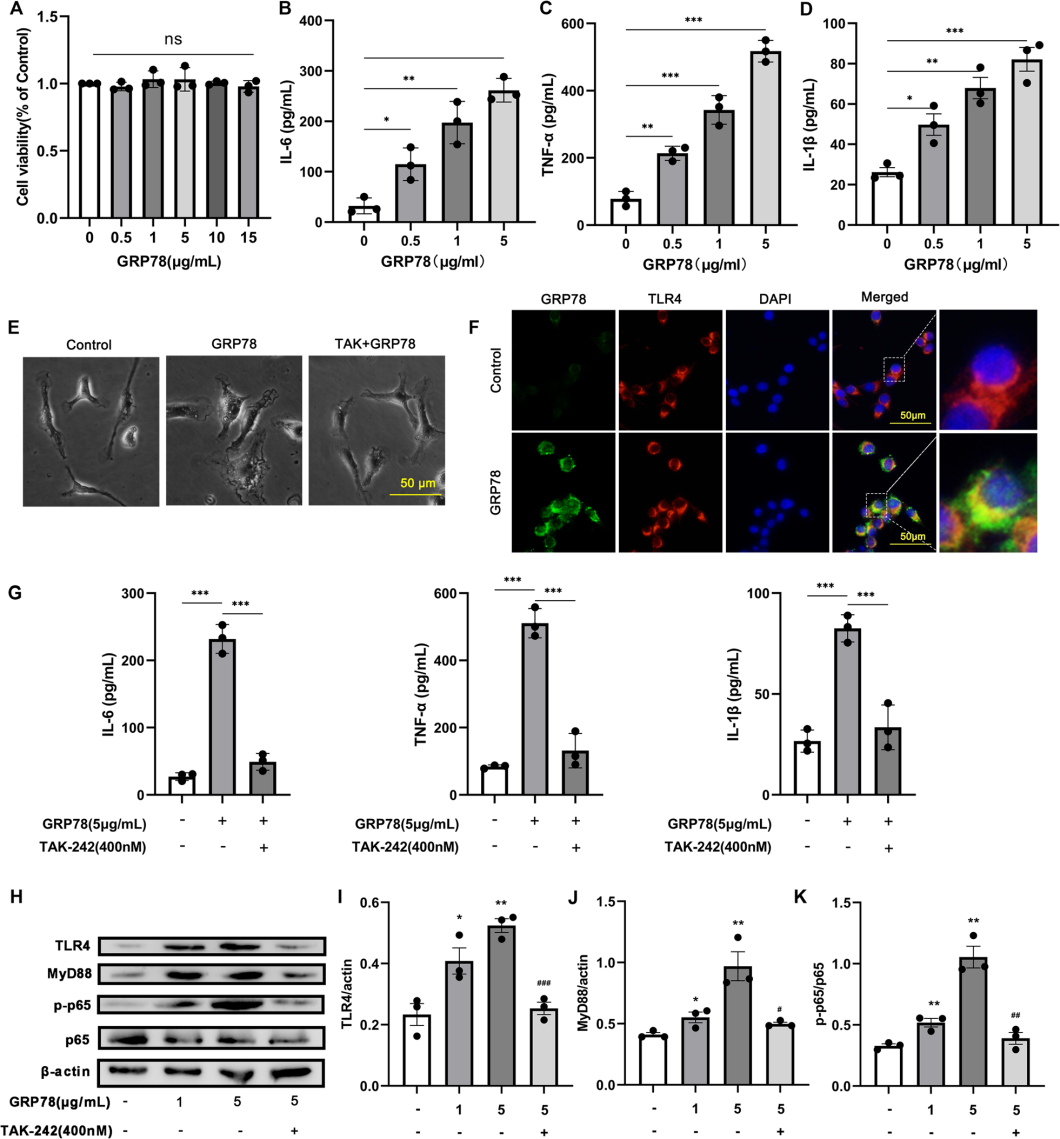
Activation of microglia induced by GRP78 via TLR4. **A** Quantitative results of cell viability detected by CCK-8 assay for primary microglia incubated with different concentrations of GRP78 (*n* = 3). Data are mean ± SEM. **B–D** Comparisons of IL-6 (**B**), TNF-α (**C**) and IL-1β (**D**) in the supernatant of primary microglia incubated with different concentrations of GRP78 (*n* = 3). **E** Changes in the morphology of primary microglia upon GRP78 treatment. **F** Double immunofluorescence for GRP78 (green) and TLR4 (red) of BV2 cells. **G** Comparisons of IL-6, TNF-α, and IL-1β in the supernatant of primary microglia upon GRP78 stimulation with or without TAK-242 pretreatment (*n* = 3). *p < 0.05, **p < 0.01, ***p < 0.001, one-way ANOVA and Tukey’s multiple comparisons test. **H**: Representative western blotting images showing levels of TLR4, MyD88, p-p65, p65, and β-actin. **I–K** Densitometric quantification of TLR4/ actin (**B**), MyD88/ actin (**C**), and p-P65/ P65 (**D**) in different groups (*n* = 3). *Comparison to control, *p < 0.05, **p < 0.01. ^#^comparison to GRP78 (5 μg/ml) group, ^##^*p* < 0.01, ^###^*p* < 0.001. Data are mean ± SEM. *n* = 3. One-way ANOVA and Tukey’s multiple comparisons test

To explore the effect of GRP78 on microglia, we stimulated primary microglia with mouse recombinant GRP78 protein for 24 h. The results showed that cell viability was not influenced by GRP78 at a concentration < 15 μg/ml, and the levels of IL-6, TNF-α and IL-1β in the supernatants increased in a GRP78 concentration-dependent manner (Fig. 4A–D).

Microglia treated by GRP78 exhibited a “fried egg” morphology, with flattened and thickened membrane processes and a larger cell body (Fig. 4E) [29]. TAK-242 (TLR4 inhibitor) pretreatment restored the morphological changes induced by GRP78 (Fig. 4E). Immunofluorescence staining of microglia confirmed co-localization of GRP78 with TLR4 (Fig. 4F). In addition, GRP78-induced increases of IL-6, TNF-α, and IL-1β level were significantly suppressed by TAK-242 (Fig. 4G).

Next, we examined whether TLR4/MyD88/NFκB cascade was activated by GRP78 in microglia. Western blot showed that TLR4/MyD88 protein expression, and NF-κB p-p65/p65 ratio were significantly increased by GRP78 treatment in BV2 cells (Fig. 4H–K), which were suppressed by TAK-242 pretreatment. These results suggest that GRP78-induced microglial activation is mediated by TLR4/MyD88/NF-κB p65 pathway.

### GRP78 influences microglia polarization and enhances their migration and phagocytosis

**Fig. 5:**
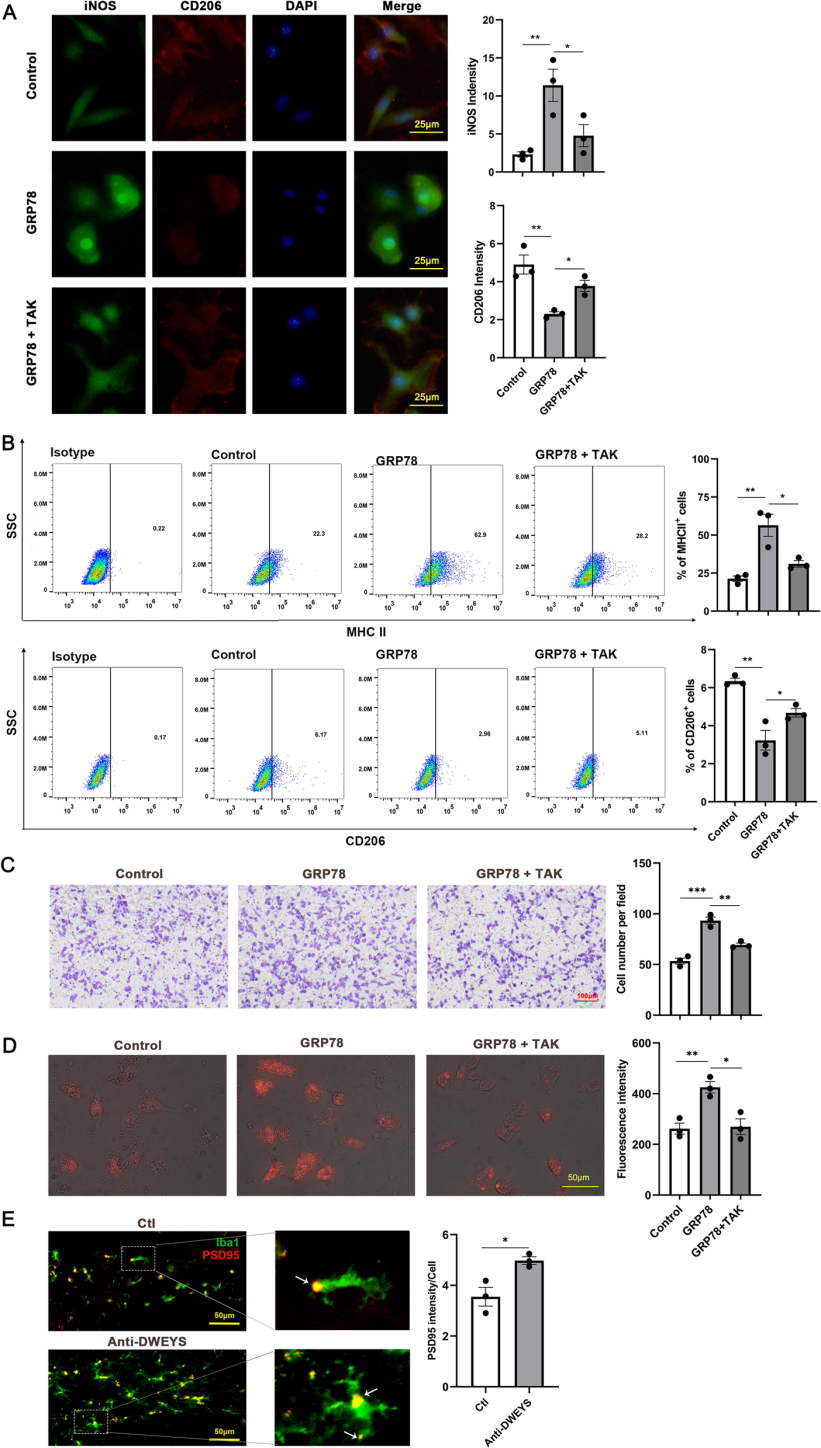
Pro-inflammatory polarization, migration, and phagocytosis of microglia induced by GRP78. **A** Left, immunofluorescence staining for iNOS, CD206, and DAPI in primary microglia upon GRP78 stimulation with or without TAK-242 treatment. Right, intensity statistics of iNOS and CD206 (*n* = 3). Data are mean ± SEM. **B** Quantification of MHC II^+^ and CD206^+^ cell upon GRP78 stimulation with or without TAK-242 treatment (*n* = 3). **C** Transwell results showed that GRP78 induced more cell migration to the lower chamber and TAK-242 had an inhibitory effect (*n* = 3). **D** Immunofluorescence assessment demonstrated the phagocytosis of fluorescent bioparticles by different groups of primary microglia (*n* = 3). **E** Left, representative images of microglia (Iba1^+^, green) containing PSD95 puncta (red) in the hippocampus from Ctl and anti-DWEYS mice. Right, quantification of PSD95^+^ puncta in microglia. *p < 0.05, **p < 0.01; ***p < 0.001, *t* test

To clarify whether GRP78 influences microglia polarization, we examined the pro-inflammatory (iNOS and MHC II) and anti-inflammatory polarization markers (CD206) in primary microglia. Microglia were divided into control, GRP78, and GRP78 + TAK groups. Results of immunofluorescence assays showed that GRP78 (5 μg/ml) induced microglia to express more iNOS and less CD206, whereas these effects were blocked by TAK-242 (Fig. 5A). Similarly, flow cytometry revealed that the percentage of MHC II^+^ cells was increased, while the percentage of CD206^+^ cells was decreased by GRP78 treatment, and TAK-242 blocked the effect of GRP78 (Fig. 5B).

Transwell assay showed that GRP78 dramatically enhanced microglia migration during 24 h of treatment, and TAK-242 repressed the migration of microglia (Fig. 5C). Fluorescence-labeled zymosan particles were used to test phagocytosis in primary microglia. More zymosan particles were found in the microglia after GRP78 treatment, indicating an increase of phagocytic activity. Also, TAK-242 pretreatment markedly suppressed the GRP78-induced microglial phagocytosis (Fig. 5D). Furthermore, we examined the phagocytosis of microglia in the hippocampus of anti-DWEYS mice by double immunofluorescence for Iba1 (green) and PSD95 (red, Fig. 5E). Microglia from anti-DWEYS mice contained more internalized PSD95 puncta than those from Ctl mice. Thus, microglia in the anti-DWEYS mice may phagocytose more synaptic puncta.

### Rapamycin reversed the neuropathic changes caused by anti-DWEYS IgG

**Fig. 6:**
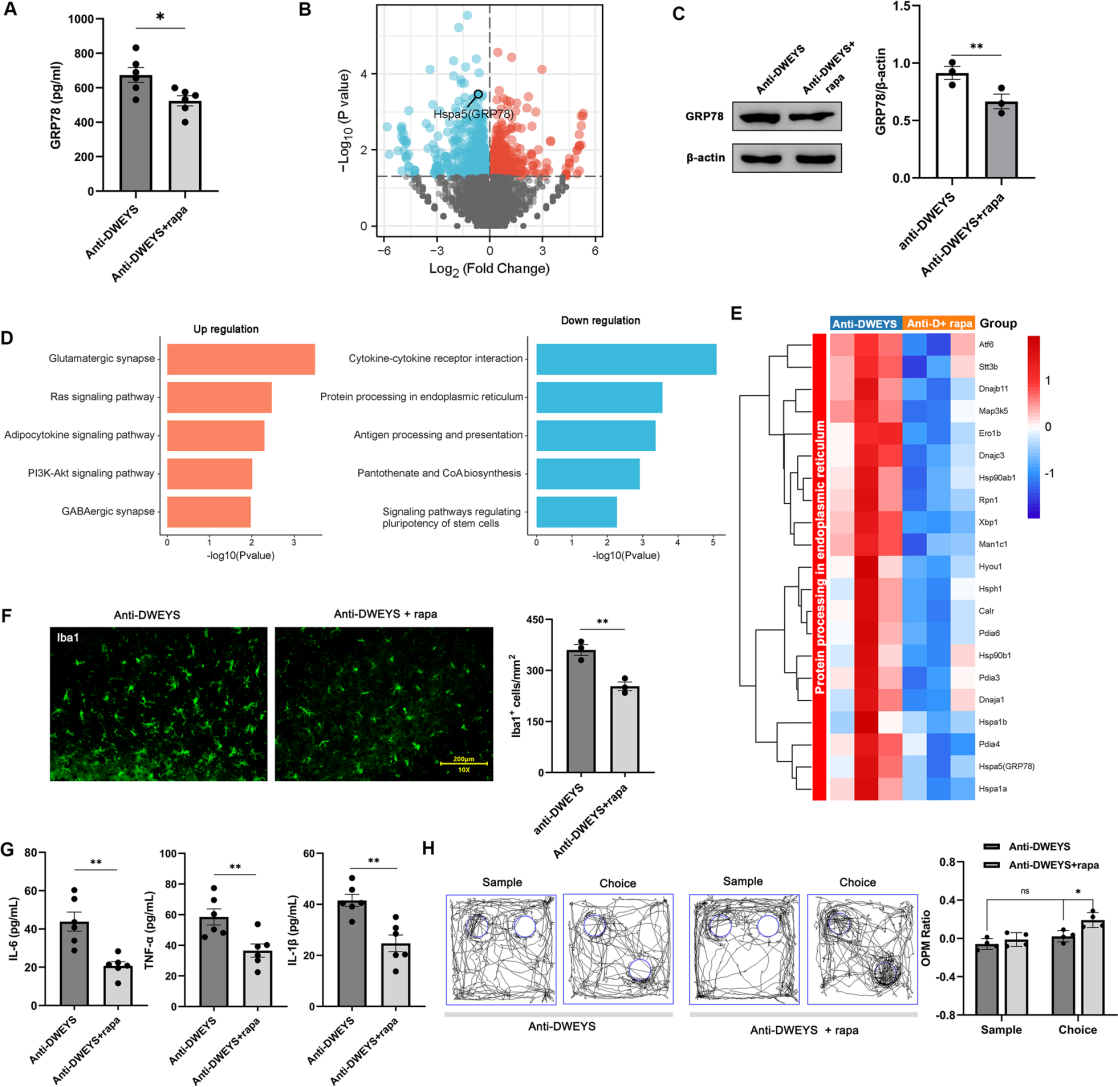
Effects of rapamycin (rapa) on the neuropathic changes in anti-DWEYS mice. **A** Comparisons of GRP78 level in the supernatant of N2a cells upon anti-DWEYS IgG stimulation with or without rapa pretreatment (*n* = 6). **B** Volcano plot showing the DEGs between anti-DWEYS and anti-DWEYS + rapa groups. **C** Left, representative western blotting images of GRP78 in anti-DWEYS and anti-DWEYS + rapa mice. Right, densitometric quantification of GRP78 in different groups (*n* = 3). **p < 0.01, *t* test. **D** KEGG pathway analysis of DEGs in up-regulated and down-regulated expression between anti-DWEYS and anti-DWEYS + rapa groups. **E** Heatmap showing the expression patterns of down-regulated DEGs in “protein processing in endoplasmic reticulum pathway”. **F** Left, immunofluorescence staining for Iba-1 in the hippocampus. Right, quantification of Iba-1^+^ cells. **G** Comparisons of IL-6, TNF-α, and IL-1β from brain homogenates in different groups (*n* = 6). **H** Performance in the OPM task of anti-DWEYS and anti-DWEYS + rapa mice (*n* = 4). Data are mean ± SEM. ns, non-significant; *p < 0.05; **p < 0.01; ***p < 0.001; *t* test

Because previous studies have shown that rapamycin can modulate neuronal ER stress [30–32], we examined whether rapamycin could reverse the neuropathic changes caused by anti-DWEYS IgG. In vitro experiments of cultured N2a cells showed that the level of GRP78 in the supernatant elevated by anti-DWEYS IgG treatment was inhibited by rapamycin (Fig. 6A). RNA-Seq analysis on the hippocampus of the anti-DWEYS mice revealed 367 up-regulated genes and 523 down-regulated genes including GRP78 (Hspa5) induced by rapamycin treatment (Fig. 6B). Western blot analysis confirmed a reduction of GRP78 expression in the hippocampus of anti-DWEYS mice treated by rapamycin (Fig. 6C). KEGG pathway analysis revealed that the “protein processing in endoplasmic reticulum” pathway was enriched in the down-regulated DEGs (Fig. 6D). Figure 6E shows that the increase or decrease of DEG expression level in “protein processing in endoplasmic reticulum” pathway was reversed by rapamycin. Microglial activation and the level of IL-6, TNF-α, and IL-1β were also significantly suppressed (Fig. 6F–G). Furthermore, the spatial memory impairments of anti-DWEYS mice were ameliorated by rapamycin (Fig. 6H).

## Discussion

In this study, we confirmed that anti-DWEYS IgG caused spatial memory impairment and microglial activation in mice. RNA-Seq analysis showed that anti-DWEYS IgG promotes protein processing in endoplasmic reticulum including GRP78 expression in hippocampal neurons. In vitro experiment demonstrated that GRP78 activated microglia, and enhanced their migration and phagocytosis via TLR4/MyD88/NFκB p65 pathway. Rapamycin reversed protein processing in endoplasmic reticulum, and inhibited neurons from producing and releasing GRP78 accompanied by weakened microglial activation and improved spatial memory impairment in the model mice. These results suggest that GRP78 may act as a mediator of the neuron–microglia crosstalk, and be a potential therapeutic target for NPSLE.

GRP78 is a molecular chaperone located on the ER membrane of all eukaryotic cells. When cells undergo ER stress, GRP78 dissociates from the ER, migrates to the cell membrane, or even is released outside the cell. Some studies showed that circulating GRP78 levels were significantly elevated in patients with myositis, obesity, and diabetes [33, 34]. An increase of GRP78 level in the brain was also found in ALS, AD, PD, ischemic stroke, as well as traumatic brain injury (TBI) [19]. In this study, we, for the first time, found an increase of CSF GRP78 level in patients with NPSLE, especially those with anti-DWEYS-associated neuropsychiatric manifestations. Serum GRP78 level was also elevated in our NPSLE cohort, but did not correlate with CSF GRP78 level, indicating that central and peripheral GRP78 may be generated independently.

Anti-dsDNA antibodies have been a hallmark of SLE. Previous studies in lupus nephritis (LN) have shown that anti-dsDNA antibodies can induce ER stress pathways in human mesangial cells [17, 35]. In CNS, a subset of the anti-dsDNA antibodies can bind to the open configuration of the NMDAR, and augment NMDAR-mediated synaptic potentials [36]. Excessive NMDAR activation can disrupt neuronal calcium balance, provoking ER stress [37]. Thus, anti-dsDNA antibodies may trigger ER stress response in NPSLE. To prove this possibility, we transferred anti-DWEYS IgG into BALB/c mice to establish an animal model, mimicking what occurs in the clinical situation of NPSLE [8, 9]. Consistent with previous studies [8, 9], our results showed that anti-DWEYS IgG deposited in hippocampal neurons and caused spatial memory impairment. In our study, we did not observe hippocampal neuron apoptosis as reported in previous research [8, 9]. This is likely due to the fact that the dose of anti-DWEYS IgG used was not sufficient to induce apoptosis of neurons. RNA-seq analysis of hippocampal tissue showed that the highest enriched pathway of up-regulated DEGs was the “protein processing in endoplasmic reticulum” pathway, and the expression of GRP78 mRNA was significantly up-regulated. Immunohistological staining revealed more expression of GRP78 in the neurons of hippocampal than astrocytes or microglia in the anti-DWEYS IgG-transferred mice. In vitro experiments confirmed that anti-DWEYS IgG could induce the release of GRP78 from neurons. Taken together, these results provide evidence that anti-DWEYS IgG could induce neurons to release GRP78.

Except for the elevation of GRP78 in many neurological diseases [19], Kakimura’s research has confirmed that GRP78 is a potent activator of microglia [19]. Here, we observed that GRP78 stimulated primary microglia to release pro-inflammatory cytokines and promoted microglia-mediated phagocytosis. As a type of pattern recognition receptors (PRRs), TLRs are located on microglia and recognize deleterious signals of foreign (bacterial or viral) or endogenous (DNA or RNA) origin [38]. GRP78 is a member of heat shock protein (HSP)-70 family, which can be actively secreted or passively released by damaged or dying cells into the extracellular space [39, 40]. Our results confirmed that GRP78 activated microglia through the TLR4/MyD88/NFκB p65 cascade, and a small molecule inhibitor of TLR4, TAK-242, could inhibit the pro-inflammatory polarization, migration, and phagocytosis of microglia induced by GRP78. However, there are significant differences observed between our in vitro and in vivo experiments in GRP78 mRNA and protein expression levels, as well as cytokine levels. This may be attributed to the fact that anti-DWEYS antibodies were temporally transferred to normal mice in our in vivo experiments. The antibody level in the recipient’s brain gradually decreased over time, leading to a decay in neural ER stress, GRP78 release, and microglial activation. We collected and examined brain tissue samples three days after the antibody transfer, during which behavioral experiments were conducted. As a result, the increases in GRP78 and cytokine levels were lower in the in vivo experiments compared to those observed in the in vitro experiments, where stimulation conditions could be consistently maintained.

Rapamycin, as a classical mTOR-C1 inhibitor, exhibits potent antitumor and immunosuppressive activity [41]. In clinical practice, rapamycin has been demonstrated efficacy and safety for SLE patients with renal or skin remission [42]. Previous studies have demonstrated that rapamycin can inhibit ER stress in neurons, and GRP78 is a marker protein of ER stress [32, 43]. Our in vitro experiments confirmed that rapamycin can suppress the release of GRP78 in N2a cells induced by anti-DWEYS IgG. In vivo experiments also demonstrated that rapamycin can reduce the levels of GRP78 in the brain tissue of anti-DWEYS mice. Therefore, rapamycin has a potential inhibitory effect on neural ER stress. Additionally, we found that GRP78 may be an important mediator of crosstalk between neurons and microglia. GRP78 released by neurons undergoing ER stress can activate microglia and promote the release of pro-inflammatory cytokines, as well as enhance their phagocytic function, which causes spatial memory impairments. Increasing evidence suggests that the release of pro-inflammatory cytokines by activated microglia can impair neurogenesis and neuroexcitation [10, 25], and mediate excessive hippocampal synaptic pruning, which causes structural damage to hippocampal neurons [8, 44, 45]. These have been demonstrated to be important neuropathological mechanisms of spatial memory impairments in NPSLE mice [8, 46, 47]. Importantly, in addition to its ability to inhibit ER stress in neurons, rapamycin may also simultaneously suppress the inflammatory activation of microglia [29]. The multiple effects of rapamycin may contribute to its significant capability in improving the behavioral abnormalities in the early phase of anti-DWEYS mouse model. Overall, our results confirm rapamycin’s protective effect in NPSLE.

## Conclusions

Taken together, we demonstrated that anti-DWEYS IgG can stimulate neurons to release GRP78, which is involved in the formation of neuropsychiatric effects on a rodent model by activating microglia in the hippocampus via the TLR4/MyD88/NFκB signaling pathway. And rapamycin plays a protective role in this model. Therefore, our findings suggest that GRP78 is a neuron–microglia crosstalk and potential therapeutic target for NPSLE.

## Data Availability

All data generated or analyzed during the course of this study are included in the published article and its supplementary documents, and are available upon reasonable request to the corresponding author.

## Abbreviations

NPSLE: Neuropsychiatric systemic lupus erythematosus
SLE: Systemic lupus erythematosus
GRP78: Glucose regulatory protein 78
CSF: Cerebrospinal fluid
CNS: Central nervous system
BBB: Blood–brain barrier
NMDAR: N-Methyl-d-aspartic acid receptor
AD: Alzheimer’s disease
ALS: Amyotrophic lateral sclerosis
PD: Parkinson’s disease
ER: Endoplasmic reticulum
IL: Interleukin
TNF: Tumor necrosis factor
ELISA: Enzyme-linked immunosorbent assay
OPM: Object place memory
KEGG: Kyoto Encyclopedia of Genes and Genomes
DEGs: Differentially expressed genes

## References

[1] Tsokos GC (2011). Systemic lupus erythematosus. N Engl J Med.

[2] Hanly JG (2014). Diagnosis and management of neuropsychiatric SLE. Nat Rev Rheumatol.

[3] Jianing W, Jingyi X, Pingting Y (2022). Neuropsychiatric lupus erythematosus: Focusing on autoantibodies. J Autoimmun.

[4] Browne K, Zhang E, Sullivan JK (2021). Lupus-prone B6.Nba2 male and female mice display anti-DWEYS reactivity and a neuropsychiatric phenotype. Brain Behav Immun.

[5] Chan K, Nestor J, Huerta TS (2020). Lupus autoantibodies act as positive allosteric modulators at GluN2A-containing NMDA receptors and impair spatial memory. Nat Commun.

[6] Bosch X, Ramos-Casals M, Khamashta MA (2012). The DWEYS peptide in systemic lupus erythematosus. Trends Mol Med.

[7] DeGiorgio LA, Konstantinov KN, Lee SC (2001). A subset of lupus anti-DNA antibodies cross-reacts with the NR2 glutamate receptor in systemic lupus erythematosus. Nat Med.

[8] Nestor J, Arinuma Y, Huerta TS (2018). Lupus antibodies induce behavioral changes mediated by microglia and blocked by ACE inhibitors. J Exp Med.

[9] Chang EH, Volpe BT, Mackay M (2015). Selective impairment of spatial cognition caused by autoantibodies to the N-Methyl-D-Aspartate receptor. EBioMedicine.

[10] Borst K, Dumas AA, Prinz M (2021). Microglia: immune and non-immune functions. Immunity.

[11] Bartels T, De Schepper S, Hong S (2020). Microglia modulate neurodegeneration in Alzheimer’s and Parkinson’s diseases. Science.

[12] Hetz C, Saxena S (2017). ER stress and the unfolded protein response in neurodegeneration. Nat Rev Neurol.

[13] Yin Y, Sun G, Li E (2017). ER stress and impaired autophagy flux in neuronal degeneration and brain injury. Ageing Res Rev.

[14] Ferreiro E, Oliveira CR, Pereira C (2004). Involvement of endoplasmic reticulum Ca2+ release through ryanodine and inositol 1,4,5-triphosphate receptors in the neurotoxic effects induced by the amyloid-beta peptide. J Neurosci Res.

[15] Demuro A, Parker I (2013). Cytotoxicity of intracellular aβ42 amyloid oligomers involves Ca2+ release from the endoplasmic reticulum by stimulated production of inositol trisphosphate. J Neurosci.

[16] Paula-Lima AC, Adasme T, SanMartín C (2011). Amyloid β-peptide oligomers stimulate RyR-mediated Ca2+ release inducing mitochondrial fragmentation in hippocampal neurons and prevent RyR-mediated dendritic spine remodeling produced by BDNF. Antioxid Redox Signal.

[17] Zhang H, Zhao C, Wang S (2015). Anti-dsDNA antibodies induce inflammation via endoplasmic reticulum stress in human mesangial cells. J Transl Med.

[18] Gerakis Y, Hetz C (2018). Emerging roles of ER stress in the etiology and pathogenesis of Alzheimer’s disease. FEBS J.

[19] Ghemrawi R, Khair M (2020). Endoplasmic reticulum stress and unfolded protein response in neurodegenerative diseases. Int J Mol Sci.

[20] Kakimura J, Kitamura Y, Taniguchi T (2001). Bip/GRP78-induced production of cytokines and uptake of amyloid-beta(1–42) peptide in microglia. Biochem Biophys Res Commun.

[21] The American College of Rheumatology nomenclature and case definitions for neuropsychiatric lupus syndromes. Arthritis Rheum. 1999;42(4):599–608. 10.1002/1529-0131(199904)42:4. 10.1002/1529-0131(199904)42:4<599::AID-ANR2>3.0.CO;2-F 10211873

[22] Matus S, Burgos PV, Bravo-Zehnder M (2007). Antiribosomal-P autoantibodies from psychiatric lupus target a novel neuronal surface protein causing calcium influx and apoptosis. J Exp Med.

[23] Kowal C, DeGiorgio LA, Nakaoka T (2004). Cognition and immunity; antibody impairs memory. Immunity.

[24] Bravo-Zehnder M, Toledo E, Segovia-Miranda F (2015). Anti-ribosomal P protein autoantibodies from patients with neuropsychiatric lupus impair memory in mice. Arthritis Rheumatol (Hoboken, NJ).

[25] Monje ML, Toda H, Palmer TD (2003). Inflammatory blockade restores adult hippocampal neurogenesis. Science.

[26] Murgoci AN, Duhamel M, Raffo-Romero A (2020). Location of neonatal microglia drives small extracellular vesicles content and biological functions in vitro. J Extracell Vesicles.

[27] Hirohata S, Arinuma Y, Yanagida T (2014). Blood-brain barrier damages and intrathecal synthesis of anti-N-methyl-D-aspartate receptor NR2 antibodies in diffuse psychiatric/neuropsychological syndromes in systemic lupus erythematosus. Arthritis Res Ther.

[28] Tsai SY, Hayashi T, Harvey BK (2009). Sigma-1 receptors regulate hippocampal dendritic spine formation via a free radical-sensitive mechanism involving Rac1xGTP pathway. Proc Natl Acad Sci U S A.

[29] Wang J, Yang C, Hou X (2021). Rapamycin modulates the proinflammatory memory-like response of microglia induced by BAFF. Front Immunol.

[30] Fan B, Sun YJ, Liu SY (2017). Neuroprotective strategy in retinal degeneration: suppressing er stress-induced cell death via inhibition of the mTOR signal. Int J Mol Sci.

[31] Cho BJ, Hwang JS, Shin YJ (2019). Rapamycin rescues endoplasmic reticulum stress-induced dry eye syndrome in mice. Invest Ophthalmol Vis Sci.

[32] Wan H, Wang Q, Chen X (2020). WDR45 contributes to neurodegeneration through regulation of ER homeostasis and neuronal death. Autophagy.

[33] Xiao F, Tan J-Z, Xu X-Y (2015). Increased levels of HSPA5 in the serum of patients with inflammatory myopathies–preliminary findings. Clin Rheumatol.

[34] Nourbakhsh M, Sharifi R, Heydari N (2021). Circulating TRB3 and GRP78 levels in type 2 diabetes patients: crosstalk between glucose homeostasis and endoplasmic reticulum stress. J Endocrinol Invest.

[35] Hirabayashi Y, Oka Y, Tada M (2007). A potential trigger of nephritogenic anti-DNA antibodies in lupus nephritis. Ann N Y Acad Sci.

[36] Faust TW, Chang EH, Kowal C (2010). Neurotoxic lupus autoantibodies alter brain function through two distinct mechanisms. Proc Natl Acad Sci U S A.

[37] Costa RO, Lacor PN, Ferreira IL (2012). Endoplasmic reticulum stress occurs downstream of GluN2B subunit of N-methyl-d-aspartate receptor in mature hippocampal cultures treated with amyloid-β oligomers. Aging Cell.

[38] Li L, Acioglu C, Heary RF (2021). Role of astroglial toll-like receptors (TLRs) in central nervous system infections, injury and neurodegenerative diseases. Brain Behav Immun.

[39] Qu J, Tao XY, Teng P (2017). Blocking ATP-sensitive potassium channel alleviates morphine tolerance by inhibiting HSP70-TLR4-NLRP3-mediated neuroinflammation. J Neuroinflammation.

[40] Dos Santos RS, Veras FP, Ferreira DW (2020). Involvement of the Hsp70/TLR4/IL-6 and TNF-α pathways in delayed-onset muscle soreness. J Neurochem.

[41] Benjamin D, Colombi M, Moroni C (2011). Rapamycin passes the torch: a new generation of mTOR inhibitors. Nat Rev Drug Discov.

[42] Peng L, Wu C, Hong R (2020). Clinical efficacy and safety of sirolimus in systemic lupus erythematosus: a real-world study and meta-analysis. Ther Adv Musculoskelet Dis..

[43] Thon M, Hosoi T, Yoshii M (2014). Leptin induced GRP78 expression through the PI3K-mTOR pathway in neuronal cells. Sci Rep.

[44] Lu L, Wang H, Liu X (2021). Pyruvate kinase isoform M2 impairs cognition in systemic lupus erythematosus by promoting microglial synaptic pruning via the β-catenin signaling pathway. J Neuroinflammation.

[45] Han X, Xu T, Ding C (2022). Neuronal NR4A1 deficiency drives complement-coordinated synaptic stripping by microglia in a mouse model of lupus. Signal Transduct Target Ther.

[46] Makinde HM, Winter DR, Procissi D (2020). A novel microglia-specific transcriptional signature correlates with behavioral deficits in neuropsychiatric lupus. Front Immunol.

[47] Kim J, Jeon SG, Jeong HR (2022). L-Type Ca(2+) channel inhibition rescues the LPS-induced neuroinflammatory response and impairments in spatial memory and dendritic spine formation. Int J Mol Sci.

